# Severe Reactions to Rituximab in Children: A Cohort Study of Rituximab-Induced Serum Sickness and Anaphylaxis

**DOI:** 10.3390/children13040442

**Published:** 2026-03-24

**Authors:** Camille Feltesse, Jean-François Delisle, Roxane Labrosse, Colette Deslandres, Nadia Roumeliotis, Jean Jacques De Bruycker, Véronique Phan, Thomas Pincez, Yves Pastore

**Affiliations:** 1 Department of Pediatric Hematology and Oncology, Centre Hospitalier Universitaire de Sherbrooke, University of Sherbrooke, Sherbrooke, QC J1H 5H3, Canada; 2 Department of Pharmacy, Centre Hospitalier Universitaire Sainte Justine, University of Montreal, Montreal, QC H3T 1C5, Canada; 3 Division of Allergy and Immunology, Department of Pediatrics, Centre Hospitalier Universitaire Sainte Justine, University of Montreal, Montreal, QC H3T 1C5, Canada; 4 Division of Gastroenterology, Hepatology and Nutrition, Department of Pediatrics, Centre Hospitalier Universitaire Sainte Justine, University of Montreal, Montreal, QC H3T 1C5, Canada; 5 Department of Pediatric Intensive Care, Centre Hospitalier Universitaire Sainte Justine, University of Montreal, Montreal, QC H3T 1C5, Canada; 6 Division of Nephrology, Department of Pediatrics, Centre Hospitalier Universitaire Sainte Justine, University of Montreal, Montreal, QC H3T 1C5, Canada; 7 Department of Pediatric Hematology and Oncology, Centre Hospitalier Universitaire Sainte Justine, University of Montreal, Montreal, QC H3T 1C5, Canada

**Keywords:** rituximab, serum sickness, anaphylaxis, pediatric, immunology

## Abstract

**Highlights:**

**What are the main findings?**

- Rituximab-induced serum sickness is a rare infusion-related reaction, but it seemed more frequent in patients treated for an autoimmune condition.
- The typical triad of fever, rash, and arthralgia was frequent in children with rituximab-induced serum sickness.

**What is the implication of the main finding?**

- Rituximab-induced serum sickness should be promptly recognized in children, as evolution was favorable with treatment.
- Treatment alternatives should be pursued after rituximab-induced serum sickness, given the risk of severe reactions if rituximab is rechallenged.

**Abstract:**

Background/Objectives: Severe infusion-related reactions to rituximab are rare; we aim to extend our knowledge about them in children, focusing on rituximab-induced serum sickness (RISS) and anaphylaxis. Methods: We conducted a monocentric retrospective study on children and adolescents who received rituximab. Patients were defined as having RISS if they had fever and at least rash and/or arthralgia, 1 to 30 days following infusion, and without another diagnosis to explain symptoms. Anaphylaxis was defined according to the diagnostic criteria proposed by the World Allergy Organization. Results: 1534 rituximab infusions in 391 patients were analyzed. Seven patients developed RISS; all received rituximab for an autoimmune disease, including four for immune thrombocytopenia (ITP). Six patients had fever, rash, and arthralgia. C-reactive protein or sedimentation rate was increased in all patients, and complement was decreased in 83%. Evolution was favorable within a few days with corticosteroids and/or intravenous immunoglobulins. Rituximab was reinfused in one patient, which resulted in an immediate anaphylactoid reaction. Lower doses of rituximab were less likely to induce RISS. RISS was associated with a greater chance of achieving ITP remission. Seven patients developed anaphylaxis; five successfully received further infusions using desensitization protocols. Conclusions: RISS in children is a severe complication of rituximab infusion. Our study suggests that it may be more frequent in individuals treated for autoimmune conditions, especially ITP. The classical triad of fever, rash, and arthralgia appeared to be frequently present, and biological inflammation and/or low complement can further support the diagnosis. In contrast to anaphylaxis, where rituximab may be safely rechallenged upon desensitization protocol, treatment alternatives should be pursued in patients experiencing RISS, given the higher risk of severe RISS recurrence.

## 1. Introduction

Rituximab (Rituxan^®^) is a chimeric monoclonal antibody with human IgG1 immunoglobulin constant regions and variable regions from an anti-CD20 murine antibody. In children, the FDA approved its use for granulomatosis with polyangiitis, microscopic polyangiitis, and, more recently, for CD20-positive diffuse large B-cell lymphoma, Burkitt lymphoma, and mature B-cell acute leukemia. Rituximab is also used off-label for other disorders involving CD20-positive cells, including numerous autoimmune disorders, glomerulonephritis, uncontrolled Epstein–Barr Virus (EBV) replication in immunosuppressed patients, post-transplant lymphoproliferative disorders, and suppression of allo-antibody production in specific cases such as humoral rejection of solid organ transplant. A retrospective study found that the use of rituximab for off-label indications in the United States increased from 1.2% in 2009 to 55.6% in 2017 [1].

Rituximab is associated with a high risk of infusion-related reaction (IRR); up of 77% of IRR arise during or after the first infusion [2]. IRRs are usually mild and transient, but severe reactions may occur. In 2021, a study on a large pediatric cohort reported an incidence of 3.6% for anaphylaxis and 0.6% for Rituximab-Induced Serum Sickness [3]. IgE and non-IgE-mediated reactions are associated with flushing, pruritus, urticaria, shortness of breath, wheezing, hypotension, and even life-threatening anaphylaxis; they are due to mast cell and basophil degranulation leading to massive histamine, leukotriene, and prostaglandin release. RISS is a rare immune complex-mediated hypersensitivity disease (type III reaction); its pathogenesis is still unclear, but it may be associated with the development of anti-rituximab antibodies. RISS typically presents as a triad associating fever, rash, and arthralgia, starting one to two weeks after infusion [4].

Although anaphylaxis is usually well recognized by physicians, RISS, in contrast, may be unrecognized, as it may be confused with infection or be associated with primary disease complications. Only a few case reports describing pediatric patients with RISS have been published [5,6,7,8,9,10,11].

This study aims to extend our knowledge of severe IRR to rituximab in children, focusing on RISS. The study also aimed at estimating the incidence of severe IRRs to rituximab (CTCAE v5.0 grade ≥ 3 [12]); we thus collected data on reported cases of anaphylaxis during the study period.

## 2. Materials and Methods

### 2.1. Inclusion Criteria

We conducted a single-center retrospective cohort study of pediatric patients who received rituximab at Centre Hospitalier Universitaire (CHU) Sainte-Justine, a tertiary and leading pediatric center in the province of Quebec (Canada), between 1 May 2014 and 31 December 2021. We included patients aged 0 to 20 years at the first rituximab infusion. Data was collected retrospectively from our electronic medical records (Chartmaxx^®^ software implemented in May 2014, version 2.0, rebranded as Quanum Enterprise Content Solutions by Quest Diagnostics, Secaucus, NJ, USA).

A list of all rituximab infusions administered at CHU Sainte Justine during the study period was obtained through the pharmacy’s database, and every patient file was reviewed. To detect potential RISS or anaphylaxis cases, we searched for hospitalizations immediately following infusion (for anaphylaxis) or in the 30-day period following infusion (for RISS). RISS and anaphylaxis patients were also detected through archives by crossing “serum sickness” or “anaphylaxis” codes with the “rituximab” code.

The following data were collected from patient charts: patients’ characteristics, including age, sex, weight, and height at the time of each infusion, rituximab indication, doses administered, delay from infusion to reaction, treatment administered, and clinical and biological presentation and outcome of the reaction.

### 2.2. Data Classification

Rituximab doses were recorded for each patient and classified into four categories: 375 mg/m^2^ (±10%), 500 mg/m^2^ (±10%), dose lower than 337.5 mg/m^2^, and higher than 550 mg/m^2^. Patients receiving more than one infusion who may have received different doses were also categorized as “mixed doses”.

Rituximab indications were separated into six main categories: autoimmunity, solid organ graft rejection, EBV infection, dysimmunity following hematopoietic stem cell transplantation, lymphomas, and other causes.

### 2.3. Rituximab Administration

Rituximab was administered using standard institutional procedure in agreement with the manufacturer’s recommendation. If the first infusion was well tolerated, subsequent infusions were administered with a quicker infusion rate. Standard premedication consisted of intravenous (IV) diphenhydramine and oral acetaminophen.

### 2.4. RISS Definition

Although a peer-approved definition of serum sickness is not available in the literature, RISS diagnosis is based on the association of a compatible temporal course (symptoms occurring between 1 and 21 days after the last infusion) and symptoms typically including fever, rash, and polyarthralgia or arthritis [4,13].

Considering the existing literature, we chose to use the following criteria to include patients in the RISS subgroup:

1. Temporality: symptoms developing 1 to 30 days following the rituximab infusion;
2. Symptoms: fever and at least one of the two following: rash and/or arthralgia;
3. No other confirmed diagnosis to explain the symptoms (infection, disease flare-up, other causes).

The study was approved by the Institutional Review Board of the Sainte Justine Hospital.

### 2.5. Anaphylaxis Definition

The diagnostic criteria proposed by the World Allergy Organization in 2020 [14] were used to classify patients in the anaphylaxis subgroup.

Per these criteria, anaphylaxis is highly likely when either one of the following two criteria is fulfilled:

1. Acute onset of an illness (minutes to several hours) with simultaneous involvement of the skin, mucosal tissue, or both (e.g., generalized hives, pruritus or flushing, swollen lips-tongue-uvula), and at least one of the following:
  - Respiratory compromise (e.g., dyspnea, wheeze-bronchospasm, stridor, reduced peak expiratory flow, hypoxemia);
  - Reduced blood pressure or associated symptoms of end-organ dysfunction (e.g., hypotonia [collapse], syncope, incontinence);
  - Severe gastrointestinal symptoms (e.g., severe crampy abdominal pain, repetitive vomiting), especially after exposure to non-food allergens.
2. Acute onset of hypotension ^a^ or bronchospasm ^b^ or laryngeal involvement ^c^ after exposure to a known or highly probable allergen ^d^ for that patient (minutes to several hours), even in the absence of typical skin involvement.
  ^a^ Hypotension is defined as a decrease in systolic BP greater than 30% from that person’s baseline, OR
  Infants and children under 10 years: systolic BP less than (70 mmHg + [2 × age in years])
  Adults and children over 10 years: systolic BP less than <90 mmHg.
  ^b^ Excluding lower respiratory symptoms triggered by common inhalant allergens or food allergens perceived to cause “inhalational” reactions in the absence of ingestion.
  ^c^ Laryngeal symptoms include stridor, vocal changes, and odynophagia.
  ^d^ An allergen is a substance (usually a protein) capable of triggering an immune response that can result in an allergic reaction. Most allergens act through an IgE-mediated pathway, but some non-allergen triggers can act independently of IgE (for example, via direct activation of mast cells).

### 2.6. Statistical Analysis

Categorical variables were compared using Fisher’s exact test, and continuous variables were analyzed using the Mann–Whitney U test. A two-sided *p* value < 0.05 was considered statistically significant.

## 3. Results

Between 1 May 2014 and 31 December 2021, 391 patients aged 0 to 20 years received rituximab with a total of 1534 rituximab infusions. The mean number of infusions per patient was 3.9 infusions, with a median of 4 infusions per patient (interquartile range (IQR) = 2–5).

Indications for rituximab are shown in Table 1. Rituximab was administered for autoimmune disorders in 61.1% of patients, including neurologic, nephrologic, hematologic, and gastrointestinal diseases, and complex multisystemic disorders. Other indications for rituximab included lymphomas, EBV reactivation, and post-allo-hematopoietic stem cell transplantation (HSCT) dysimmunity.

### 3.1. Rituximab-Induced Serum Sickness

Seven out of 391 patients presented symptoms and criteria for RISS (1.8% of patients); patient characteristics and RISS details are described in Supplementary Table S1. Mean age at RISS was 12.3 years; 57.1% of patients were female.

All seven patients received premedication before their rituximab infusions; five patients were on steroids at the time of infusion. The mean time from rituximab to RISS was 9 days, ranging from 6 to 12 days. Time to reaction was shorter when RISS occurred after the second rituximab infusion (3 patients; mean 7.7 days) than after the first infusion (4 patients; mean 10.8 days), although the difference was not statistically significant.

Most patients (6/7) presented with the classical triad of fever, rash, and arthralgia; cutaneous manifestation was absent in only one patient. All patients had an elevated sedimentation rate and/or C-reactive protein; complement was measured in 6 patients and decreased in the majority (5/6).

RISS was recognized in all but one patient. Three patients required Intensive Care Unit (ICU) admission due to hemodynamic instability, two of whom were treated with epinephrine. No deaths occurred. RISS was treated with corticosteroids in 5 patients, along with intravenous immunoglobulins (IVIG) in 2 patients, while one received IVIG only. Symptomatology resolved in all patients within 4 days. An RISS diagnosis was made retrospectively in only one patient, but evolution did not differ from that of other patients, and symptoms resolved spontaneously after 3 days. Mean duration of RISS was 4 days, ranging from 3 to 6 days.

All seven patients with RISS had received rituximab for an autoimmune disorder. The incidence of RISS was 2.9% among patients treated for autoimmune disorders, compared with 0% among those treated for other indications. Among the seven patients with RISS, four had immune thrombocytopenia (ITP), all achieving partial (1/4) or complete (3/4) remission. The only patient who achieved partial remission had secondary ITP in the context of complex dysimmunity. During our study period, fifteen other patients received rituximab for chronic ITP, and 66% (10/15) achieved partial or complete remission. Comparing patients with ITP who developed RISS to those who did not, RISS was therefore associated with a greater chance of achieving partial or complete remission (*p* = 0.033, Risk ratio = 3, CI 1.47–6.14). Initial disease followed its usual course for other non-ITP patients, with successive flare-ups interspersed with remission periods. Overall, ITP patients who received high doses of rituximab had a higher rate of partial or complete remission (8/9 vs. 6/10); however, the difference was not statistically significant (*p* = 0.3). We also compared the overall risk of developing RISS in all patients who received only one or multiple standard doses (375 mg/m^2^) with those who received one or multiple high doses (500 mg/m^2^). Patients who received standard dose(s) appeared to be at lower risk of developing RISS (risk ratio 0.14, CI 0.03–0.74, *p* = 0.016).

Rituximab was rechallenged in the only patient whose RISS diagnosis was made retrospectively. She presented an immediate reaction with dyspnea, fever, vomiting, and rash, which resolved after treatment with hydrocortisone, diphenhydramine, and dimenhydrinate. Rituximab was then permanently discontinued. Unfortunately, none of our patients were tested for the presence of anti-rituximab antibodies.

### 3.2. Anaphylaxis

Seven patients in our cohort developed an anaphylactic reaction to rituximab (1.8% of patients); patient characteristics and anaphylaxis details are described in Table 2. In contrast to patients with RISS, anaphylaxis occurred similarly in patients treated for autoimmune diseases as well as for other indications.

All seven patients received premedication before rituximab infusions. Three patients had an anaphylactic reaction to the first infusion. The mean time from the start of infusion to anaphylaxis was 69 min, ranging from 40 to 110 min.

Three patients presented low blood pressure or syncope; symptoms responded quickly to treatment, and no patient needed to be transferred to the intensive care unit. One patient received only antihistamine treatment (diphenhydramine), two patients received diphenhydramine and IV hydrocortisone, and three patients received diphenhydramine, IV hydrocortisone, and epinephrine. One patient presented with isolated low blood pressure and only received an IV bolus.

Desensitization protocols were successfully used thereafter for four patients (patients 9, 10, 12, and 14). Patient 11 received two subsequent doses without desensitization protocols, but with a slower rate of infusion; treatment was well tolerated.

Allergy skin tests were not available in our patients’ electronic charts.

## 4. Discussion

Rituximab is a chimeric monoclonal antibody targeting CD20. It leads to B-cell depletion by a combination of immune-mediated mechanisms, including antibody-dependent cell-mediated cytotoxicity, complement-dependent cytotoxicity, and direct effects induced by CD20 ligation [15]. As such, it is used to treat a wide array of conditions in which B cells or CD20-bearing cells are involved.

Similar to reports in the adult population, IRRs in children are frequent but mostly mild (CTCAE v5.0 grade 1 or 2), occurring in 15.7% to 60% [3,16,17,18,19]. Grade ≥ 3 IRRs such as RISS and anaphylaxis are reported in less than 5% of patients. In line with previous findings [3], 3.6% of children and adolescents in our cohort developed RISS or anaphylaxis.

IRR to rituximab mostly occur within the first infusion and have been reported in up 88.3% of IRRs in children and adolescents [3,16]. Most of these IRRs are caused by a cytokine release triggered by B-cell destruction and macrophage activation during clearance of immune complexes. These cytokine-release reactions are usually mild, can be attenuated with the use of premedication, and resolve after slowing or temporarily interrupting the infusion [20].

Out of the fourteen patients with grade ≥ 3 IRR described in our study, half reacted to the first infusion (50%); the other half reacted following subsequent infusions. For IgE-mediated anaphylaxis, initial sensitization is required; severe reactions are therefore usually observed after a subsequent exposure to the allergen. Other non-IgE-mediated pathways can also lead to anaphylaxis, making a severe reaction also possible during or after the first infusion [21].

### 4.1. Rituximab-Induced Serum Sickness

In contrast to anaphylactic reactions and IRR, serum sickness is an immune complex-mediated type III delayed hypersensitivity reaction, and its physiopathology is less well understood. Following immunization with rituximab, antibodies begin to develop 7 to 14 days later and form immune complexes with the antigen. Immune complexes then deposit in tissues and lead to complement activation and recruitment of inflammatory cells. Considering this mechanism of action, RISS can occur after the first exposure. Previous studies have reported RISS after a single rituximab infusion in 25 to 54.1% of cases [4,13].

Although the size of our cohort remains limited, it is yet the largest to be reported. We identified ten other cases of RISS in pediatric patients from case reports found through a literature review. For description purposes, we will refer below to these ten patients from the literature as patients A to J. Six received rituximab for ITP, and four for nephrotic syndrome (Supplementary Table S2). All ten patients received a dose of 375 mg/m^2^ of rituximab. No patient needed to be transferred to the ICU. Time to RISS onset after the rituximab infusion was reported for only five patients and ranged from 8 to 10 days (mean 9.4 days); time to onset was reported as “7 to 14 days” for two additional patients.

No ICU admissions have been reported in the literature in pediatric patients. In contrast, 3 of our 7 RISS patients presented with hypotension requiring ICU admission, two of whom received epinephrine. Selection bias leading to under-detection of mild cases not requiring hospitalization cannot be excluded; however, the incidence of RISS in our cohort was similar to previous reports.

Rituximab was re-challenged in two patients reported. Patient F was able to complete a full treatment of four infusions without further reactions; there was no data available about premedication. Patient H presented with rash and arthralgia after the second infusion, and anaphylaxis after the third infusion; rituximab was then discontinued. Patient H received premedication with chlorphenoxamine and acetaminophen before each infusion.

Anti-rituximab antibodies were detected in four patients reported: patient A (27 days after RISS), G (2 months after RISS), I (57 days after RISS), and J (during RISS).

Among the six patients treated for ITP, the quality of response to rituximab was available in five patients: two failed to respond to rituximab (patients B and C), while three had a transient or complete response (patient D relapsed after 3 months, and patients E and F were still in remission 6 and 19 months after RISS, respectively).

Among patients treated for nephrotic syndrome, patient G obtained remission, and patient J relapsed 35 days after RISS. Evolution was not disclosed for patients H and I.

The first case of RISS was reported in 2001 in an adult patient, 10 days after receiving rituximab for refractory autoimmune polyneuropathy [22]. Since then, RISS has mainly been reported through retrospective case reports. The RISS incidence of 1.8% in our study was higher than the 0.6% incidence previously reported in another study, and similar to the rate of severe anaphylactic reactions [3]. However, the low incidence of RISS cases reported in both studies may account for this variability.

In 2015, a systematic review identified 33 RISS cases from 25 articles [4]; 6 cases occurred in children or adolescents and are also part of our literature review. A report from the French Pharmacovigilance Database from 1998 to 2016 identified 37 cases, with a median age of 38 years [IQR 29–53] [13]. While females may be more affected than males, the difference was not as marked in children compared with adults (58.8% of 17 combined pediatric patients vs. 70–78.2% of adult patients). Time to onset of RISS was longer following the first dose of rituximab when compared with subsequent doses in both the systematic review and the French Pharmacovigilance database (10 days vs. 4.1 days and 8 days vs. 3.3 days, respectively). RISS also occurred more rapidly after subsequent doses compared with the first doses in our cohort. Among the ten pediatric patients with RISS from our literature review, the precise time to the onset of RISS was reported for only five patients, and only one patient presented with RISS after the first infusion. Even with data collection extended to 30 days post-rituximab infusion, our findings align with previous reports, showing RISS in pediatric patients occurring 5–12 days after infusion.

RISS typically presents with a triad of symptoms including fever, arthralgia, and urticarial rash [4,13]. Such a classical triad was present in 14 of the 27 adults (51.8%) analyzed in the systematic review [4]. Combining data from our cohort and literature review, the triad of fever, rash, and arthralgia was found in 11 cases (64.7%); fever was reported in 15 patients (88.2%), arthralgia in 16 patients (94.1%), and rash in 12 patients (70.6%).

While there is no biological marker for RISS, typical findings include elevated inflammatory markers and decreased complement levels [4,13]. In our case series, systemic inflammation was found in all our RISS patients (7/7), complement consumption in 83% (5/6 tested), and proteinuria in 42.8% (3/7). Anti-rituximab antibodies, or human anti-chimeric antibodies (HACAs), were unfortunately not searched for in our cohort. According to the literature, HACAs may be found. In the 2015 systematic review, HACAs were tested in eleven patients and were positive in only six [4]. Other studies found that the development of HACAs may not necessarily be associated with the occurrence of RISS [11,23,24]. Therefore, the exact role of HACAs in the pathophysiology of RISS still remains unclear.

Although the classic triad may be more frequent in children than in adults, RISS should be considered in the differential diagnosis of any suggestive symptom developing within 21 days of rituximab infusion. The presence of biological inflammation and/or low serum complement can further increase the likelihood of RISS.

Patients receiving rituximab for autoimmune disease may be at higher risk for RISS. All seven cases of RISS in our cohort occurred in patients treated for autoimmune disorders. Similarly, 85% of RISS cases reported by Karmachary et al. had received rituximab for autoimmune conditions [4]. Bayer et al. also suggested an incidence of RISS at least 12 times higher in autoimmune conditions than in hematological malignancies [13]. All pediatric patients from our cohort and the literature have also been treated for an autoimmune condition, dominated by two main conditions: ITP (58.8%, *n* = 10), followed by nephrotic syndrome (23.5%, *n* = 4). In our cohort, 21.1% of ITP children treated with rituximab developed RISS, a rate that is almost twice that of most previous reports (12.5%) [9]. We hypothesize that the use of a high rituximab dose in up to 42.5% of our patients may explain such a greater rate. Indeed, use of a higher rituximab dose appeared to be associated with a greater risk of developing RISS in our cohort. While there was no correlation between the dose used in ITP patients and remission, the development of RISS was associated with a greater chance of obtaining a partial or complete remission in our cohort. Such findings should be taken cautiously, given the small size of our cohort, and could not be replicated through our literature review. Such an association should therefore be evaluated in further prospective studies to exclude false positives due to confounding factors. The possible underlying mechanism is unclear.

Systemic lupus erythematosus (SLE) may also be associated with the risk of RISS. In the French pharmacovigilance report, 11 of the 28 RISS patients treated for autoimmune diseases had SLE [13]. In our pediatric cohort, only 1 out of 25 lupus patients developed RISS. RISS recognition in patients with rheumatologic conditions such as lupus might be particularly challenging, as symptomatology may also mimic a disease flare-up.

RISS may be associated with higher risks of subsequent reactions if rituximab is reinfused. Other case series have indeed reported severe anaphylactoid/anaphylaxis reactions or RISS recurrence after rituximab re-infusion [6,25,26]. The only patient from our cohort who received a rituximab infusion after RISS presented an immediate anaphylactoid infusion reaction; rituximab was then permanently discontinued. While the rare occurrence of successful desensitization to rituximab after developing RISS has been reported [27], we believe that Rituximab reinfusion after RISS should be avoided whenever possible.

### 4.2. Anaphylaxis

Anaphylactic reaction to rituximab infusion is a rare but life-threatening complication. Delay in appropriate treatment can be fatal; however, the case fatality for anaphylaxis is under 0.001% (all causes mixed) [28].

The incidence of anaphylaxis has been reported to be 1–4% across studies, and most often occurs following the first infusion [3,29,30,31,32]. In line with previous reports, 1.8% of our patients presented with anaphylaxis; however, only 42.8% reacted after the first dose. We found no associations of anaphylaxis risk and specific conditions.

Five of our seven patients were re-infused with rituximab after anaphylaxis, among whom four followed a desensitization protocol, and one received a slower infusion rate. Re-exposure was successful for all of them. Desensitization protocols have indeed proven effective when rituximab is reinfused in children and adolescents after developing severe hypersensitivity reactions such as anaphylaxis [33,34,35,36,37].

### 4.3. Strengths and Limitations

We focused on RISS incidence and characteristics in a pediatric population treated with rituximab and were able to find seven patients with RISS among 392 patients who received rituximab for various conditions. The sample size evaluated is one of the strengths of our study, describing the largest retrospective cohort on RISS in children and providing detailed data about RISS for each patient. It provides clinically relevant information by identifying that RISS occurring only in patients with an autoimmune condition may be related to the rituximab dose received and associated with a higher likelihood of response. Furthermore, our patient with RISS who developed anaphylaxis after reinfusion supports the few data suggesting avoiding reusing rituximab after RISS.

However, our study has several limitations. First, our cohort being retrospective, there is a risk of misclassification bias due to incompleteness in the medical records. Second, our methods did not allow detection of mild to moderate RISS cases not requiring hospitalization. However, such cases typically resolve without specific treatment, limiting the clinical impact of potential under-detection. Because all hospital admissions within 30 days of each rituximab infusion were reviewed, incomplete documentation of more symptomatic cases is unlikely, although treatment at another hospital cannot be excluded. Third, further pediatric RISS cases were collected from existing literature, so publication bias cannot be excluded. Fourth, as no universally accepted definition of RISS exists to date, we defined RISS using criteria derived from the existing literature while acknowledging that alternative causes could account for similar symptoms. Although infectious causes were investigated, undetected infections cannot be excluded. Misclassification as an autoimmune disease flare cannot be ruled out, particularly in patients treated for autoimmune conditions other than ITP, as ITP flares typically do not present with rash and arthralgia. Ideally, the diagnosis of RISS should be based upon a clinical score or more robust system; further studies are therefore needed to search for a validated scoring system. Fifth, anti-rituximab antibodies may play a role in the pathogenesis of serum sickness; however, we were unable to assess this, as testing was not performed in our cohort. Finally, the cohort size of pediatric patients remains limited. RISS and anaphylaxis being relatively rare complications, conclusions drawn from association and risk factors analyses are limited.

## 5. Conclusions

RISS is a rare, delayed hypersensitivity reaction induced by deposits of immune complexes in tissues, usually occurring within 7 to 14 days following rituximab infusion. In children, RISS seems to be more frequent when rituximab is administered for an autoimmune condition, especially immune thrombocytopenic purpura. RISS should be promptly recognized, since although symptoms usually resolve within a few days with corticosteroids, they can be severe and associated with hypotension and shock requiring ICU admission. Diagnosis is made based on clinical presentation and timeframe.

## Figures and Tables

**Table 1:** Patient sex, rituximab indication, and dose(s) received.

Parameters	Cohort *N* = 391		RISS *N* = 7		Anaphylaxis *N* = 7	
	*N*	% of Total Cohort	*N*	% of Specific Parameter	*N*	% of Specific Parameter
SEX						
Female	203	51.9	4/203	2.0	4/203	2.0
Male	188	48.1	3/188	1.6	3/188	1.6
						
INDICATION						
Auto-immunity	239	61.1	7/239	2.9	0	0
Renal	70	17.9	0	0	0	0
Neurologic	60	15.3	1/60	1.7	2/60	3.3
Hematologic	39	10.0	4/39	10.3	2/39	5.1
ITP	19	4.9	4/19	21.1	0	0
Gastro-intestinal	9	2.3	0	0	0	0
Complex	53	13.3	1/53	1.9	0	0
Lupus	25	6.4	1/25	4.0	0	0
Other ^a^	9	2.3	1/9	11.1	0	0
Graft rejection	14	3.6	0	0	1/14	7.1
(solid organ transplant)						
EBV infection	85	21.7	0	0	2/85	2.4
Secondary PTLD	10	2.6	0	0	1/10	10.0
Post-allo-HSCT dysimmunity	25	6.4	0	0	0	0
Lymphoma	29	7.4	0	0	0	0
Other ^b^	16	4.1	0	0	0	0
						
DOSE						
375 mg/m^2^ (±10%)	289	73.9	2	0.7	4	1.4
only ^c^	219	56.0	2	0.9	4	1.8
500 mg/m^2^ (±10%)	141	36.1	5	3.5	3	2.1
only	79	20.2	5	6.3	1	1.3
Low (<337.5 mg/m^2^)	25	6.4	0	0	0	0
only	7	1.8	0	0	0	0
High (>550 mg/m^2^)	24	6.1	0	0	2	8.3
only	10	2.6	0	0	0	0
Mixed doses	79	20.2	0	0	2	2.5

EBV: Epstein–Barr Virus, PTLD: Post Transplant Lymphoproliferative disorder; HSCT: Hematopoietic Stem Cell Transplantation. ^a^ Other auto-immunity: anti-HU paraneoplastic syndrome (*n* = 1), ROHHAD syndrome (*n* = 1), APDS (*n* = 1), primary immune deficiency with TTC7A mutation (*n* = 1), post-chemotherapy auto-immunity (*n* = 1), atypical hemolytic uremic syndrome (*n* = 1), IPEX-like syndrome (*n* = 1), autoimmune myopathy (*n* = 1), tricho-hepato-enteric syndrome (*n* = 1); ^b^ Other: Hemophagocytic lymphohistiocytosis (HLH) (*n* = 4), inhibitor allo-antibodies in hemophiliac patient (*n* = 3), pre-transplant anti-HLA antibodies (*n* = 2), Pompe disease (*n* = 3), hyperhaemolysis in a sickle cell patient (*n* = 1), pre-hematopoietic stem cell transplantation conditioning (*n* = 3); ^c^ 79 patients received different doses of Rituximab throughout their treatment. The mention “only” refers to the number of patients who only received the dose mentioned and no other.

**Table 2:** Patients with anaphylaxis: patient characteristics and description of the reaction.

	8	9	10	11	12	13	14
**Patient characteristic**							
Sex	F	F	F	M	F	M	M
Diagnosis	Post-HSCT EBV reactivation	Evans syndrome—NFkB mutation	Autoimmune encephalitis	EBV-induced PTLD	Chronic inflammatory neuropathy	Autoimmune hemolytic anemia	Graft rejection (heart transplant)
Age at diagnosis (years)	3.8	8.6	12.1	16.8	1.2	16.6	0 (neonate)
**Rituximab infusion characteristics**							
Previous infusions	1	3	0	3	6	0	0
Dose (mg/m^2^)	375	375	500	375	750	375	750
Infusion to reaction delay (minutes)	60	60	100	40	110	100	15
**Clinical symptoms**							
Urticaria	Yes	Yes	Yes	No	Yes	Yes	No
Throat tightness	No	Yes	No	No	Yes	Yes	No
Respiratory	Yes	Yes	Yes	No	No	Yes	No
Gastro-intestinal	No	Yes	No	No	No	No	Yes
Hypotension or syncope	No	Yes	No	Yes	No	No	Yes
**Treatment**							
Diphenhydramine	Yes	Yes	Yes	No	Yes	Yes	Yes
Anti-H2	No	Yes	No	No	No	No	No
Epinephrine	No	Yes	Yes	No	No	Yes	No
Hydrocortisone	Yes	Yes	Yes	No	No	Yes	Yes
Acetaminophen	No	No	No	No	No	No	No
Other	No	Bolus Ventolin	Bolus	Bolus × 2 ^b^	No	No	Gravol
**Reaction duration**	13 h	3 h and 25 min ^a^	80 min	30 min × 2 ^b^	1 h	2 h and 30 min	30 min

F: Female. M: Male. HSCT: Hematopoietic Stem Cell Transplantation. EBV: Epstein–Barr Virus. PTLD: Post-Transplant Lymphoproliferative Disease. ^a^ on and off, due to trying to restart infusion. ^b^ first reaction lasted 30 min, and reoccurred after restarting infusion, needing another bolus.

## Data Availability

The original contributions presented in this study are included in the article/Supplementary Material. Further inquiries can be directed to the corresponding author.

## Supplementary Materials

The following supporting information can be downloaded at: https://www.mdpi.com/article/10.3390/children13040442/s1. Table S1: Patients with rituximab-induced serum sickness (RISS): patient characteristics and description of the reaction. Table S2: Patients with RISS from the literature: patient characteristics and description of the reaction.

## Abbreviations

The following abbreviations are used in this manuscript:

CHU	Centre Hospitalier Universitaire
CTCAE	Common Terminology Criteria for Adverse Events
EBV	Epstein–Barr Virus
FDA	Food and Drug Administration
HACA	Human Anti-Chimeric Activity
HSCT	Hematopoietic Stem Cell Transplantation
ICU	Intensive Care Unit
Ig	Immunoglobulin
IQR	Interquartile Range
IRR	Infusion-Related Reactions
ITP	Immune Thrombocytopenia
IV	Intravenous
IVIG	Intravenous Immunoglobulins
RISS	Rituximab-Induced Serum Sickness
SLE	Systemic Lupus Erythematosus
