# Supplementary material for: Severe Reactions to Rituximab in Children: A Cohort Study of Rituximab-Induced Serum Sickness and Anaphylaxis
Source: Children (Basel). 2026 Mar 24;13(4):442. doi: 10.3390/children13040442 (PMC13114620; doi:10.3390/children13040442)
Supplement: Supplementary file 1 [file children-13-00442-s001.zip › children-4196378-supplementary.pdf]

Supplementary Table S1. Patients with rituximab-induced serum sickness (RISS): patient characteristics and description of the reaction

	1	2	3	4	5	6	7
**Patient characteristics**							
Sex	F	F	M	M	F	F	M
Diagnosis	Secondary ITP	CIPD	Secondary ITP	Complex dysimmunity	Glomerulonephritis, TMA, lupus	ITP	ITP and APL syndrome
Age at diagnosis (years)	9.8	8.0	13.0	9.9	10.8	6.4	14.5
Age at RISS (years)	10.6	13.0	13.5	15.9	11.0	7.1	15.1
**Rituximab infusion characteristics**							
Steroids per-Rituximab	No	No	Yes	Yes	Yes	Yes	Yes
R1 dose (mg/m^2)	375	500	500	500	375	500	500
R2 dose (mg/m^2)	350	/	/	/	375	/	500
R1-R2 Interval (days)	7	/	/	/	6	/	143
**RISS timeframe**							
RISS after R1	No	Yes	Yes	Yes	No	Yes	No
RISS after R2	Yes	No	No	No	Yes	No	Yes
Time from infusion to RISS (days)	6	7	12	12	12	12	5
RISS duration (days)	3	5	4	4	6	5	3
ICU stay (days)	/	5	/	2	/	/	2
**Clinical symptoms**							
Rash	Yes	Yes	Yes	Yes	No	Yes	Yes
Fever	Yes	Yes	Yes	Yes	Yes	Yes	Yes
Arthralgia	Yes	Yes	Yes	Yes	Yes	Yes	Yes
**Biological symptoms**							
Proteinuria	Yes	Yes	No	No	Yes	No	No
High sedimentation rate and CRP	Yes	Yes	Yes	Yes	Yes	Yes	Yes
Low complement	Yes	Yes	N/A	Yes	No	Yes	Yes
**Treatment**							
IV Steroids	No	Yes	Yes	No	Yes	Yes	Yes
IV Immunoglobulin	No	Yes	Yes	Yes	No	No	No

ITP: idiopathic thrombocytopenic purpura. CIDP: chronic inflammatory demyelininating polyneuropathy. TMA: Thrombotic micro-angiopathy. APL: anti-phospholipid. R1/R2: First/second Rituximab infusion. ICU: Intensive Care Unit. CRP: C-Reactive Protein. N/A: Non Available.

Supplementary Table S2. Patients with RISS from the literature: patient characteristics and description of the reaction

Patient	A	B	C	D	E	F	G	H	I	J
Author	Goto et al. [5]	Bennett et al. [8]		Wang et al. [9]			Maeda et al. [10]	Bayram et al. [6]	Nakamura et al. [7]	Fujinaga et al. [11]
Study design	Case study	Prospective		Prospective			Case study	Case study	Case study	Retrospective
Sex	M	M	F	F	F	F	F	M	M	F
Diagnosis	ITP	ITP	ITP	ITP	ITP	ITP	NS	NS	NS	NS
Age at RISS (years)	8	12	11	14	12	12	17	7	6	N/A
Rituximab dosage (mg/m^2)	375	375	375	375	375	375	375	375	375	375
Infusions before RISS	2	2	2	2	3	1	5	1	4	3
Time from infusion to RISS	10 days	N/A	N/A	7 to 14 days	7 to 14 days	N/A	10 days	8 days	10 days	9 days
RISS duration	N/A	N/A	N/A	N/A	N/A	N/A	5 days	1 day	4 days	N/A
Rash	Yes	Yes	Yes	Yes	Yes	No	No	No	No	Yes
Fever	Yes	Yes	Yes	Yes	Yes	No	Yes	No	Yes	Yes
Arthralgia	Yes	No	Yes	Yes	Yes	Yes	Yes	Yes	Yes	Yes
Anti-rituximab antibodies	244 ng/ml	N/A	N/A	N/A	N/A	N/A	184 ng/ml	N/A	> 5000 ng/ml	1984 ng/ml
High ESR and/or CRP	N/A	N/A	N/A	N/A	N/A	N/A	No	No	No	N/A
Low complement	N/A	N/A	N/A	N/A	N/A	N/A	No	No	N/A	N/A

ITP: Idiopathic Thrombocytopenic Purpura. NS: Nephrotic Syndrome. M: Male. F: Female. N/A: Not Available. ESR: Erythrocyte Sedimentation Rate. CRP: C-Reactive Protein.
